# Editorial: Non-coding RNAs as potential therapeutics and biomarkers for human diseases

**DOI:** 10.3389/fphar.2025.1753536

**Published:** 2025-12-18

**Authors:** Margarida Gama-Carvalho, Alice Conigliaro, Chiara De Santi

**Affiliations:** 1 BioISI – Biosystems and Integrative Sciences Institute, Faculty of Sciences, University of Lisbon, Lisbon, Portugal; 2 Department of Biomedicine Neuroscience and Advanced Diagnostic, University of Palermo, Palermo, Italy; 3 School of Pharmacy and Biomolecular Sciences, Royal College of Surgeons in Ireland, Dublin, Ireland

**Keywords:** biomarkers, CircRNAs, lncRNAs, miRNAs, ncRNA, RNA therapeutics

## NcRNAs in health and disease

Once considered “junk DNA”, non-coding genomic regions have gained substantial attention over the past 2 decades (Pennisi, 2012). A very rich and diverse set of non-coding RNA (ncRNA) transcripts has been revealed thanks to technological advances in transcriptomics and bioinformatic annotation methods. In the current GENCODE release, 7,563 small non-coding RNA (sncRNA) genes and 35,899 long non-coding RNA genes (lncRNAs) are annotated in the human genome, exceeding the number of protein-coding genes by more than two-fold (Mudge et al., 2024).

The catalogue of sncRNA continues to expand, with families defined by their biogenesis, associated protein complexes and cellular functions (Shi et al., 2022). microRNAs (miRs) remain the most extensively characterized, regulating mRNA stability and translation, and influencing key biological processes, including proliferation, metabolism, differentiation, and organ development (Shang et al., 2023). Other sncRNA classes, like piRNAs, are central to genome stability and antiviral defense, or participate in RNA biogenesis and modification pathways (e.g., snoRNAs in rRNA biogenesis). Additional groups, such as tRNA-derived fragments, illustrate the increasing complexity of this regulatory layer.

LncRNAs have only recently begun to be characterized systematically (Mattick et al., 2023). Their capacity to form diverse ribonucleoprotein assemblies and act across multiple regulatory levels complicates efforts to classify them or predict function, although many operate as modulators of chromatin and transcription. A third major class – circular RNAs (circRNAs) – initially considered “splicing noise”, has emerged as a distinct group with covalently closed structures and regulatory functions increasingly recognized across physiological and pathological contexts. Predominantly generated by head-to-tail back-splicing events from “parental” gene transcripts, most circRNAs regulate gene expression by acting as sponges for proteins and miRs, although other functions have been reported (Yang et al., 2022).

**FIGURE 1:**
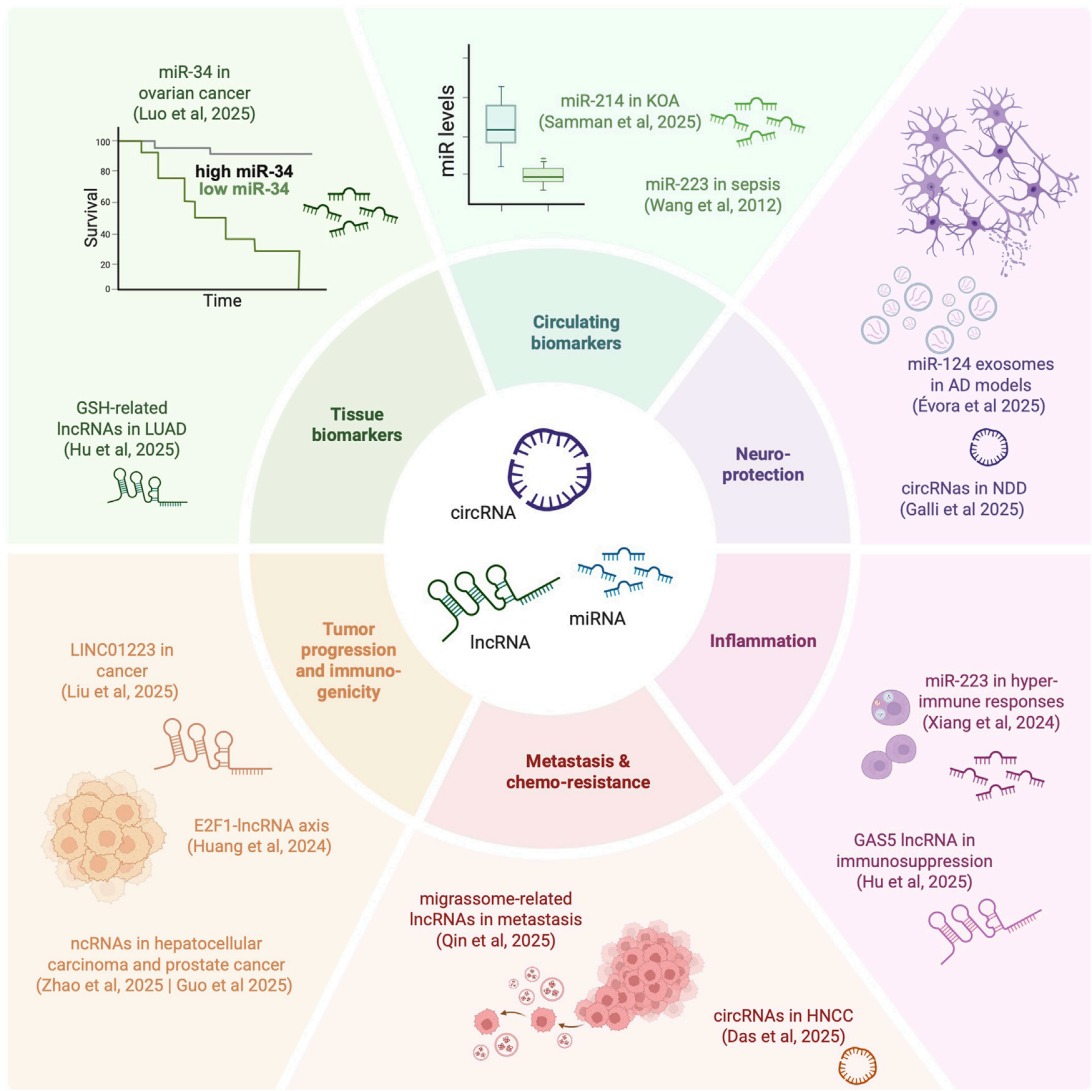
Non-coding RNAs as Potential Therapeutics and Biomarkers for Human Diseases. Different areas of impact of ncRNAs covered in this Research Topic. AD – Alzheimer’s Disease. HNCC – head and neck carcinoma. KOA – knee osteoarthritis. LUAD – lung adenocarcinoma. NDD – neurodegenerative disorders. Created in BioRender. Gama-Carvalho, M. (2025) https://BioRender.com/vdnbyzf.

Of relevance for pharmacology, specific ncRNAs show altered expression across diverse pathological contexts, and have been proposed as diagnostic or prognostic biomarkers, particularly in neurological diseases and cancer. Their presence in circulation–either freely or within extracellular vesicles - enhances their potential as minimally invasive disease monitors. Beyond biomarker applications, ncRNA actively modulate pathological processes, making them attractive therapeutic targets or agents. In cancer, for instance, ncRNAs influence oncogenic signaling and tumor-immune interactions, highlighting translational opportunities. The associate review by Guo et al. on ncRNA regulation of the PI3K/AKT/mTOR pathway in prostate cancer showcases this potential. Collectively, the articles assembled in this Research Topic provide a high-level view of a rapidly evolving field with direct relevance to disease monitoring and therapeutic development (Figure 1).

## MiRNAs take the lead

The Research Topic includes a set of original studies on miRs that illustrate their clinical and translational relevance across diverse pathological contexts. Several miRs have been proposed as biomarkers for disease severity and prognosis. A meta-analysis of tumor expression levels of the onco-suppressor miR-34 family from seven studies involving 672 women, revealed that higher expression is associated with significantly improved overall survival and progression-free survival, supporting a possible prognostic value for these miRs (Luo et al.) (Samman et al.) explore the correlation of serum levels of miR-214, a known regulator of the Wnt-signalling pathway, in patients with knee osteoarthritis (KOA), proving evidence for decreased levels compared to healthy controls. Their observations proof that this miR is functionally linked to a pathogenic signalling axis (Wnt/β-catenin) in KOA, being quantitatively associated with disease severity and structural damage, suggesting that, beyond a promising biomarker, it may be of value as a therapeutic agent too. Circulating levels of miR-223 were found to be altered in the context of sepsis (Wang et al., 2012). In a continuation of this study published as part of this Research Topic, authors now show that miR-223 participates in the regulation of autophagy in CD4^+^ T lymphocytes and potentially limits cytokine-driven hyperimmune responses, supporting its relevance as both biomarker and therapeutic target for sepsis (Xiang et al.). Finally, the work of Évora et al. explores the potential of miR-124-3p loaded exosomes, showing they exert neuro- and immune-protective effects across neurons, microglia, and astrocytes in a microfluidic Alzheimer’s disease triculture model characterized by neurodegeneration and glia-driven neuroinflammation.

Collectively, the miR studies featured in this Research Topic underscore the capacity of sncRNAs to link molecular regulation with clinically observable phenotypes. They exemplify how miRs can serve as accessible biomarkers, inform disease mechanisms and support the development of targeted therapeutic strategies across inflammatory and degenerative conditions.

## LncRNAs in tumor progression and immunogenicity

LncRNAs offer substantial opportunities in disease definition and treatment. Research in this area focuses on two main fronts: identifying novel lncRNAs within the genome and characterizing their functional mechanisms. The latter is particularly challenging, as lncRNAs can exert contrasting effects depending on cellular context and cofactors.

In this Research Topic, Curci et al. investigated lncRNA GAS5 in inflammation, showing that it influences the NF-κB pathway in a context-depending manner. GAS5 directly binds to p65, boosting NF-κB ability to bind to DNA. Notably, when glucocorticoids (GCs) are present, GAS5 reverses its role: it inhibits NF-κB DNA binding and strengthens the anti-inflammatory effects of GCs. These results identify GAS5 as an important modulator of inflammation and immune responses. as both a biomarker for GC therapy response and a potential target to improve efficacy or overcome resistance.

Another study reports original work on the use of lncRNA-based signatures in cancer prognosis and therapy. Hu et al. developed prognostic model based on glutathione (GSH) metabolism-related lncRNAs in lung adenocarcinoma. By linking GSH metabolism, lncRNA expression, and immune contexture, the model predicts patient outcomes and correlates with immune infiltration, tumour mutation burden, and drug sensitivity - supporting personalized treatment strategies.

LncRNAs have been extensively studied as regulators of cancer progression. In this Research Topic, Huang et al. reviewed the current literature on the effect of the bidirectional regulation of several lncRNAs towards the (largely) pro-oncogenic transcription factor E2F1 in different cancer types, suggesting that targeting the E2F1-lncRNA network could represent a viable therapeutic option. Liu and colleagues summarised the current literature around a novel lncRNA called LINC01123 in tumour progression, metabolism, immune escape, and resistance (Liu et al.). The function of lncRNAs in tumour immunogenicity and response to immunotherapy in hepatocellular carcinoma is the focus of another review (Zhao et al.), highlighting once again that lncRNAs do not only modulate the biology of the cancer cells their selves but also the overall tumour microenvironment, with important consequences on immune responses to tumour development and treatment outcomes.

A significant advancement in lncRNA biomarker research is their presence in extracellular vesicles (EVs), which facilitate intercellular communication. However, identifying the origin of EVs in biological fluids remains a challenge. The discovery of migrasomes - EVs formed during cell migration - helps narrow this uncertainty. Migrasomes released by cancer cells influence the tumour microenvironment, promoting migration and invasion. In hepatocellular carcinoma (HCC), researchers (Qin et al.) characterized migrasome-related lncRNAs (MRlncRNAs), revealing a new regulatory axis involving lncRNAs, migrasomes, and immune signalling. Their validated signature offers insights into tumour biology and supports MRlncRNA-targeted therapies and refined patient stratification in HCC.

Overall, studies in this Research Topic explore lncRNAs as biomarkers, prognostic tools, and therapeutic targets, emphasizing their context-dependent functions and emerging significance in extracellular vesicle-mediated communication and tumor microenvironment regulation.

## The promise of circular RNAs

CircRNAs are one of the most recent additions to the world of regulatory ncRNAs. In this Research Topic, three reviews were published highlighting their emerging potential as disease modifiers of complex neurologic disorders (Galli et al.), as therapeutic targets and biomarkers for colorectal cancer (Nambidi et al.), and as modulators of chemoresistance in head and neck squamous cell carcinoma (Das et al.). All three papers converge on the idea that while circRNAs are promising, moving from bench to bedside requires: (i) better mechanistic understanding; (ii) robust validation in clinical samples; and (iii) scalable and safe delivery platforms.

## Conclusion and future directions

Our Research Topic contribute to building knowledge around the role of ncRNAs in human disease in the hope that with increased understanding of the biology behind these fascinating molecules we will finally increase the chances of finding a therapeutic strategy for not-yet curable diseases.

The articles assembled in this Research Topic highlight the expanding roles of ncRNAs in biomarker development and therapeutic exploration. Together, they illustrate how diverse ncRNA families act at multiple regulatory levels and how these functions can be leveraged to improve diagnosis, stratification and intervention across pathological contexts. As the field advances, several priorities are emerging, namely, improving mechanistic understanding of ncRNA regulatory networks and developing standards for clinical-grade biomarker validation. Progress in delivery technologies and chemical modification strategies is also accelerating the translation of ncRNA-based therapeutics. The recently reported success of a small clinical trial using miRNA-based therapeutics to slow progression of Huntington’s disease (Dolgin, 2025) is an encouraging prospect for the future of the field. The contributions in this Research Topic exemplify the breadth of ongoing efforts and point to a growing set of opportunities for ncRNAs to inform future diagnostics and treatments.
